# Molecular subtypes in canine hemangiosarcoma reveal similarities with human angiosarcoma

**DOI:** 10.1371/journal.pone.0229728

**Published:** 2020-03-25

**Authors:** Guannan Wang, Ming Wu, Amy C. Durham, Enrico Radaelli, Nicola J. Mason, XiaoWei Xu, David B. Roth

**Affiliations:** 1 Department of Pathology and Laboratory Medicine, Raymond and Ruth Perelman School of Medicine, University of Pennsylvania, Philadelphia, PA, United States of America; 2 Illumina, San Diego, CA, United States of America; 3 Department of Pathobiology, School of Veterinary Medicine, University of Pennsylvania, Philadelphia, PA, United States of America; 4 Department of Clinical Sciences and Advanced Medicine, School of Veterinary Medicine, University of Pennsylvania, Philadelphia, PA, United States of America; University of California, Davis, UNITED STATES

## Abstract

Angiosarcoma (AS) is a rare neoplasm with limited treatment options and a poor survival rate. Development of effective therapies is hindered by the rarity of this disease. Dogs spontaneously develop hemangiosarcoma (HSA), a common, histologically similar neoplasm. Metastatic disease occurs rapidly and despite chemotherapy, most dogs die several months after diagnosis. These features suggest that HSA might provide a tractable model to test experimental therapies in clinical trials. We previously reported whole exome sequencing of 20 HSA cases. Here we report development of a NGS targeted resequencing panel to detect driver mutations in HSA and other canine tumors. We validated the panel by resequencing the original 20 cases and sequenced 30 additional cases. Overall, we identified potential driver mutations in over 90% of the cases, including well-documented (in human cancers) oncogenic mutations in *PIK3CA* (46%), *PTEN* (6%), *PLCG1*(4%), and *TP53* (66%), as well as previously undetected recurrent activating mutations in *NRAS* (24%). The driver role of these mutations is further demonstrated by augmented downstream signaling crucial to tumor growth. The recurrent, mutually exclusive mutation patterns suggest distinct molecular subtypes of HSA. Driver mutations in some subtypes closely resemble those seen in some AS cases, including *NRAS*, *PLCG1*, *PIK3CA* and *TP53*. Furthermore, activation of the MAPK and PI3K pathways appear to be key oncogenic mechanisms in both species. Together, these observations suggest that dogs with spontaneous HSA could serve as a useful model for testing the efficacy of targeted therapies, some of which could potentially be of therapeutic value in AS.

## Introduction

Angiosarcoma (AS) is a rare but deadly form of cancer, arising in several clinical situations: post-irradiation therapy (e.g. for breast cancer), in sun exposed skin, and spontaneous diseases in a variety of anatomic locations. There are no effective therapies, and the 5 year survival is less than 30% [1]. The rarity of the disease has impeded identification of therapeutic targets, especially the extremely uncommon visceral form. To overcome this problem, the Angiosarcoma Project is using a crowdsourcing approach (https://ascproject.org/home) to obtain tissue and blood samples directly from patients [2]. Even as sequencing efforts begin to identify potential therapeutic targets, the rarity of AS presents challenges to performing informative clinical trials.

We have employed a comparative oncology approach to address this problem, focusing on hemangiosarcoma (HSA), a common canine tumor that, microscopically, closely resembles human AS. HSA affects approximately 50,000 dogs per year in the US [3,4]. The disease has both visceral (commonly arises in the spleen) and cutaneous forms. Dogs most commonly present with hemoabdomen following rupture of the splenic mass, and despite splenectomy plus adjuvant chemotherapy, less than 50% survives at 6 months due to progressive pulmonary metastases. We hypothesized that the molecular drivers in canine HSA are shared with human AS. If so, HSA might present an opportunity to carry out clinical trials of targeted therapies which could inform precision therapy of HSA and perhaps AS. The canine disease provides an attractive model for clinical trials because it is both common and rapidly progressive, features which allow for rapid patient recruitment and early readouts of drug safety and efficacy.

Our initial search for actionable molecular targets in HSA involved exome sequence analysis of a cohort of 20 clinical samples (tumor and matched normal) derived from client-owned dogs seeking clinical care at Penn Vet [5]. We identified recurrent somatic mutations in *PIK3CA* (activating) and in *PTEN* (inactivating) in over half of the cases. These genomic lesions correspond to mutations previously identified in human cancer (but not reported at that time in human AS), and both are capable of activating the PI3K signaling pathway. One tumor bore an activating mutation in *PLCG1* reported in human splenic angiosarcoma [6], and a number of specimens had somatic *TP53* mutations, also reported in human AS [2,7].

Our identification of recurrent, mutually exclusive patterns of mutation in this cohort of HSA samples led us to suggest that the entity defined histopathologically as HSA might actually consist of distinct molecular subtypes. We further hypothesize that if some of these canine subtypes display presumed driver mutations present in human AS, dogs bearing these tumors could serve as natural models to test targeted therapies, with the goal of informing clinical trial design and therapy of human AS. Specifically, we envision clinical trials of targeted agents in client-owned dogs in which patients are selected for particular therapies based on molecular characterization of their tumors. Such an approach in veterinary oncology would bring the principles of precision medicine, which aims to deliver the most effective treatments based on deep patient phenotyping and has largely changed the landscape of human oncology [8].

Here we report the development of an amplicon-based next generation sequencing (NGS) panel designed to rapidly and deeply sequence HSA samples derived from routine clinical material (formalin fixed, paraffin embedded blocks, FFPE). We validated the panel by re-sequencing the 20 cases previously examined by exome sequencing, and sequenced an additional 30 HSA samples. Our results define several mutually exclusive sets of driver mutations, providing the first evidence that the disease classified histologically as HSA actually consists of distinct molecular subtypes. Comparison of our data with previously published collections of AS sequences along with new data released by the Angiosarcoma Project indicate that some molecular subtypes of HSA strongly resemble mutational patterns in a subset of AS [2] (https://ascproject.org/data-release). These data suggest that therapy of certain forms of human AS might be informed by clinical trials carried out in canine patients with HSA.

## Result

### Design and development of the canine HSA panel

Based on findings from our previous whole exome sequencing (WES) and on genomic data available for canine HSA and human AS, we developed an amplicon-based targeted resequencing next generation sequencing (NGS) assay, hereby termed the "HSA panel". The HSA panel comprises [1] candidate oncogenic driver genes, recurrently mutated genes and hotspot mutations that we and others identified in canine HSA, [2] well-established oncogenic driver genes and recurrent mutations previously reported in human AS, and [3] selected genes frequently implicated in the pathogenesis of human and canine solid tumors.

**Fig 1:**
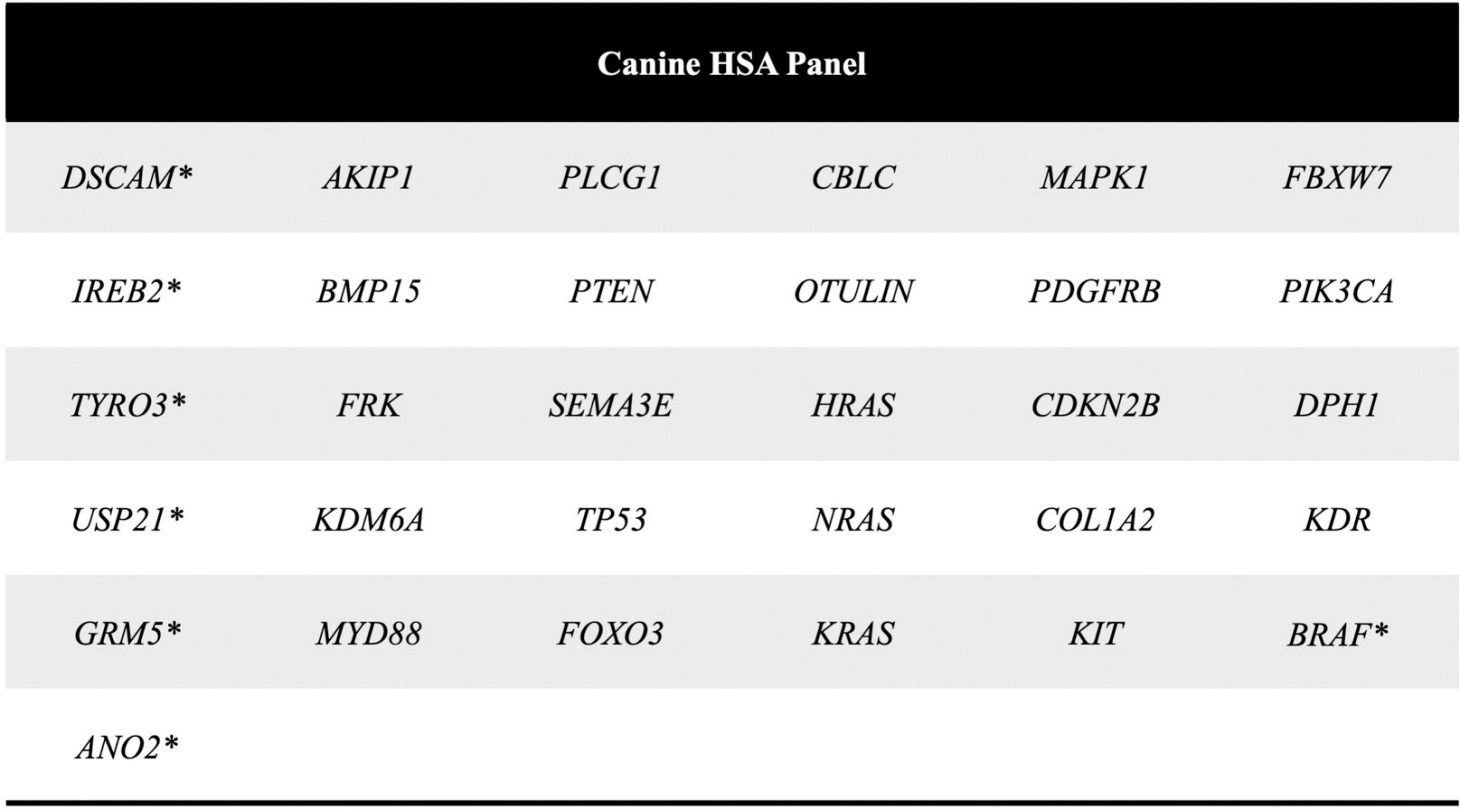
An NGS-based gene panel designed for canine hemangiosarcoma and other canine cancers, including full coding regions of 24 genes and hotspot mutations in 7 genes.

The HSA panel is designed to cover the full coding regions of 24 genes and hotspot mutations in another 7 genes (Fig 1). We employed an amplicon-based, targeted resequencing approach in which probes are designed to amplify regions of interest in the canine genome prior to making a sequencing library. Specifically, we chose to use Trueseq Custom Amplicon workflow (TSCA, Illumina), because it allows targeted resequencing at high coverage, high uniformity and high specificity. Furthermore, TSCA is compatible with FFPE samples, which makes it suitable to use on canine tissues and other veterinary clinical samples, routinely collected in formalin for histopathological evaluation. One pitfall of formalin fixation, however, is that it can cause deamination of cytidine, leading to sequence artifacts and false positive mutation calling. We therefore took advantage of the dual-pool design of TSCA that adds a second mirrored set of complementary amplicons to target both DNA strands at all targeted loci. This design requires every sample to be sequenced twice, followed by cross-comparison of variant calling from both strands, which ensures the quality of mutation calling as it eliminates false positive calls that may arise from formalin fixation. Our panel features 650 amplicons with an average length of 175 bp. Total amplicons cover a cumulative target region of 57,619 bp in the canine genome, which is greater than 99% coverage of the desired target region. The mirror set of the dual-pool design has the same parameters except that it targets the opposite strand at all loci in the canine genome.

### The HSA panel reproduces our exome sequence data and identifies additional somatic mutations

**Fig 2:**
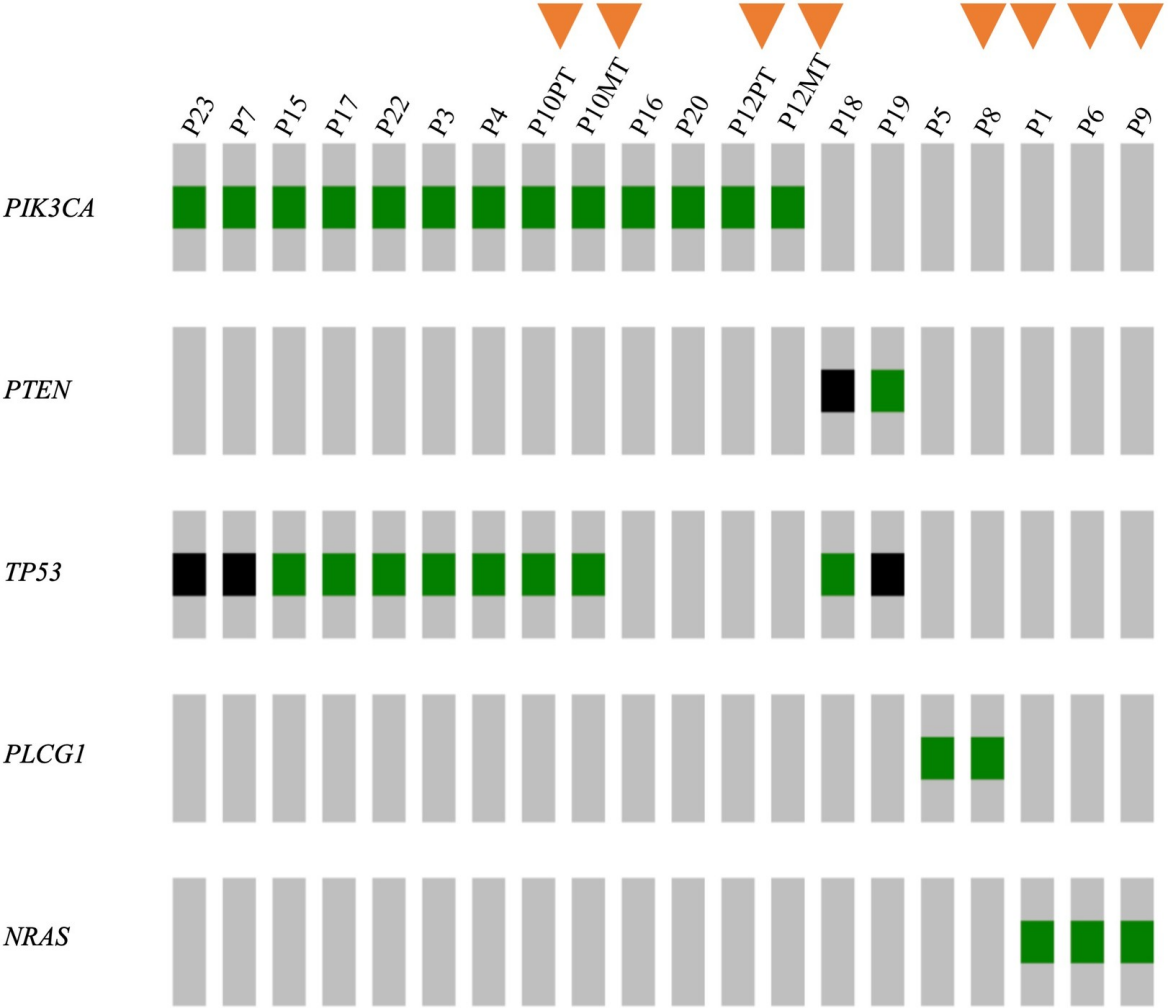
Validating the canine HSA panel with samples previously analyzed by whole exome sequencing. Candidate driver genes are shown on the left and sample (patient) ID shown on the top. PT stands for primary tumor, MT denotes metastatic tumor. Non-synonymous mutations are shown in green blocks, loss-of-function mutations (frame-shift mutations, essential splice-site variants, nonsense mutations) are shown in black blocks. Orange triangles indicates cases whose candidate driver mutations are identified by the HSA panel but not exome sequencing.

To validate our panel, we re-sequenced 20 HSA cases previously analyzed by whole exome sequencing [5]. Panel sequencing mirrored the WES findings, re-identifying all the candidate driver mutations found by WES (Fig 2). Furthermore, the HSA panel detected potential driver mutations in additional eight tumors in which WES did not detect any, as indicated by the triangles (Fig 2). In these eight tumors, not only did we observe mutations in *PIK3CA*, *PLCG1*, *TP53*, *PTEN* as previously indicated by WES, but we also identified a recurrent Q61R mutation in *NRAS* in three cases (see below for further discussion). We also observed an additional *PLCG1* alteration, G797E, a known gain-of-function mutation in human cancers [9,10]. The allele frequency (AF) of these mutations ranges from 6% to 17%, which likely explains why the *NRAS* variants were not detected by exome sequencing. The HSA panel enables detection of low AF somatic variants with high confidence, because of its deep sequence coverage, averaging over 1000X (S1 Fig), and high uniformity across target regions. We also sequenced paired primary tumor (PT) and metastatic tumor (MT) on two cases (P10 and P12), revealing high concordance of mutations in both tumor specimens from the same patient: *PIK3CA* H1047R in P10 and *PIK3CA* E726K in P12 (Fig 2 and S2 Table). Together, these data show that the HSA panel can readily detect oncogenic mutations identified by whole exome sequencing, as well as additional mutations with low AF that are missed by exome sequencing.

### The HSA panel detects putative driver mutations in over 90% of cases

**Fig 3:**
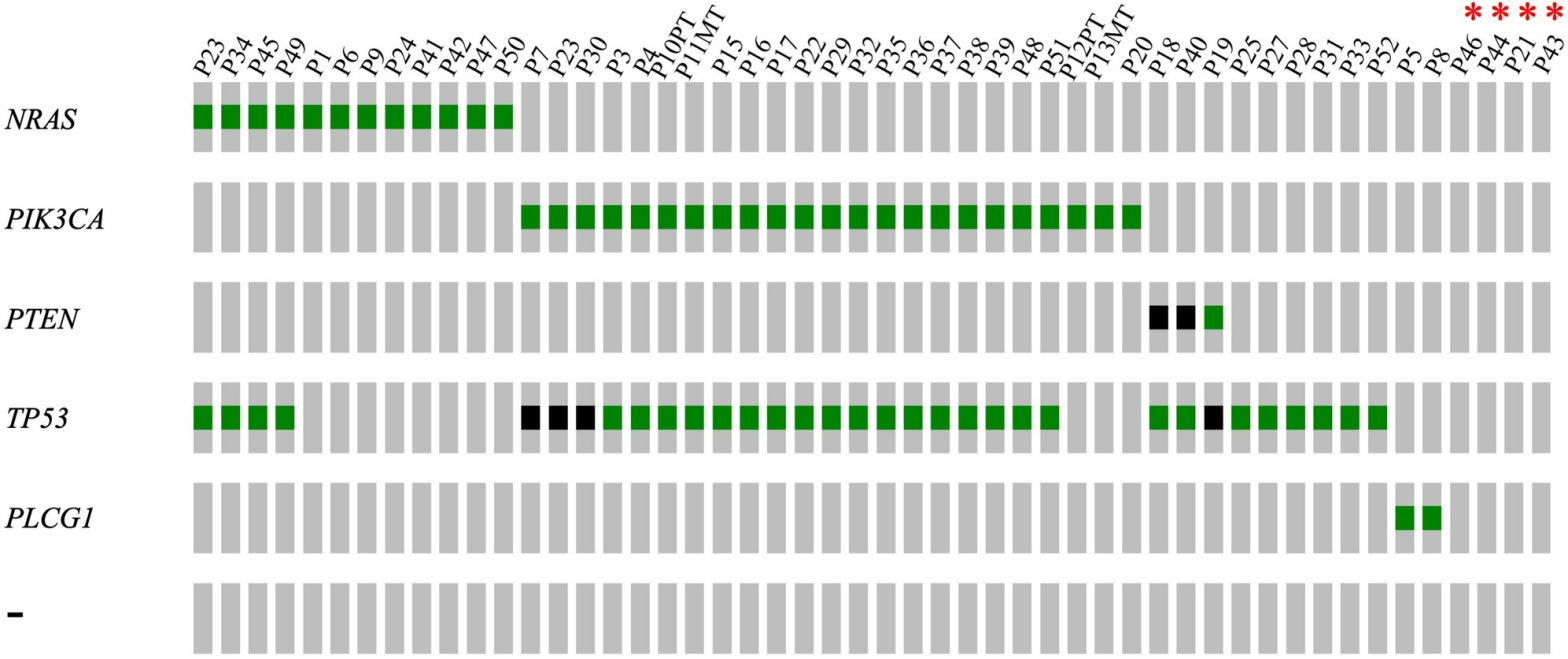
Candidate driver genes of 50 canine HSA cases determined by the HSA panel. Candidate driver genes are shown on the left and sample (patient) ID shown on the top. “NONE” group shows no potential drivers were found in those cases as indicated by the canine HSA panel. PT: primary tumor, MT: metastatic tumor. Non-synonymous mutations are shown in green blocks, loss-of-function mutations (frameshift mutations, essential splice site variants, nonsense mutations) are shown in black blocks.

Next, we employed the HSA panel to sequence an additional 30 FFPE samples of canine HSA collected through the biopsy service in the Penn Vet Diagnostic Laboratory. Candidate driver mutations identified in these cases, along with the original 20 cases (analyzed by both WES and the HSA panel) are summarized in Fig 3. Analysis of these 50 cases revealed the following recurrently mutated genes: *PIK3CA* (23/50), *PTEN* (3/50), *NRAS* (12/50), *PLCG1* (2/50) and *TP53* (33/50). Overall, we were able to identify putative driver mutations in 92% of the cases.

**Fig 4:**
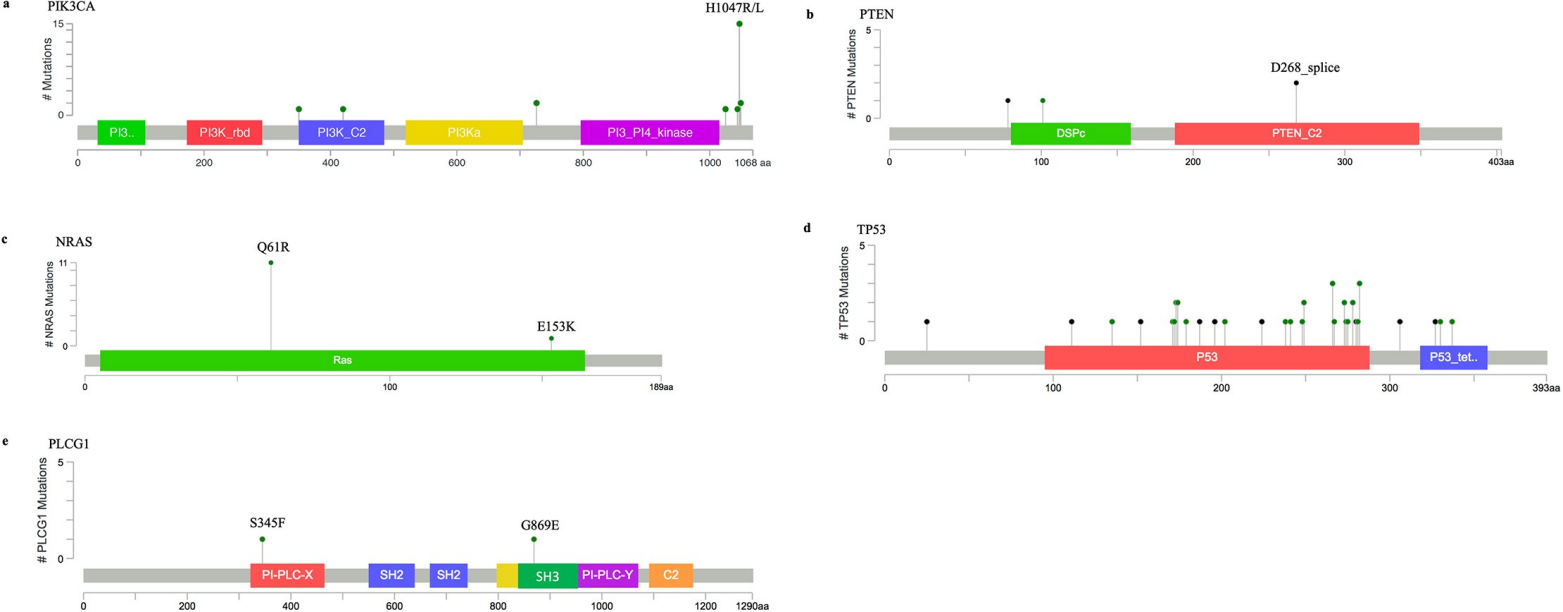
A-E: Distribution and location of the oncogenic mutations in candidate driver genes, represented in schematic diagram of the homologous mutations in human genes A. *PIK3CA*; B. *PTEN*; C. *NRAS*; D. *TP53*; E. *PLCG1*. The x axis represents domain structure and amino acid positions of the human genes, and the y axis represents the number of times each mutation was identified in our cohort.

The most frequent mutations were activating *PIK3CA* mutations (46% of cases). Most of the tumors bearing *PIK3CA* mutations also had inactivating *TP53* mutations. In addition to H1047R, D350G and E726K mutations that we previously identified through WES, we found other nonsynonymous changes in *PIK3CA*, such as E726K (2/50), G1049R (2/50), N1044K (1/50), C420R (1/50), T1025A (1/50), shown in detail in Fig 4A and S1 Table. All of these *PIK3CA* mutations have been described previously in human cancers, and most have been characterized as activating oncogenic mutations [11–13].

We observed *PTEN* mutations in three cases, including a frameshift mutation T56fs (homologous to T78fs in human *PTEN*) in P18, and recurrent identical splice site variants in P19 and P40 (D246_splice site variant, corresponding to D268_splice site variant in human *PTEN*) (Fig 4B). These recurrent splice site mutations, possibly leading to frameshift or premature truncation of *PTEN* and corresponding to the most common *PTEN* hotspot mutation, K267 frameshift. Position K267 resides in the calcium-binding 3 domain and truncation/frameshift mutations at K267 is sufficient to block *PTEN* membrane localization [11]. The nature of the alterations in *PTEN* led us to believe that they are inactivating loss-of-function mutations, which are likely oncogenic and operate in parallel with *PIK3CA* gain-of-function mutants to elevate PI3K signaling and contribute to tumorigenesis [14].

*NRAS* mutations were observed in 12/50 cases (24%). All *NRAS* mutant cases except for one harbor the same well-established oncogenic activating mutation, Q61R [15] (Fig 4C). The remaining *NRAS* mutation, E153K, Fig 4C, has not been observed in human cancer (https://cancer.sanger.ac.uk/cosmic/gene/analysis?ln=*NRAS*) [16], so its impact on tumorigenesis is yet to be determined and will be discussed below. Unlike the *PIK3CA*/*PTEN* group, most *NRAS* mutant cases lack *TP53* mutations.

In six cases (12%), we found only *TP53* mutations. These include missense mutations and a variety of truncating mutations. Most of the *TP53* alterations [35/40] are located in the DNA-binding domain and are loss-of-function mutants (Fig 4D and S2 Table). Interestingly, a few *TP53* mutations are gain-of-function mutations and associated with genome-wide instability which leads to tumorigenesis [5]. In two cases, we found *PLCG1* mutations, S273F and G797E, both corresponding to well-characterized oncogenic activating mutations in human cancers [9,17,18] (Fig 4E).

**Fig 5:**
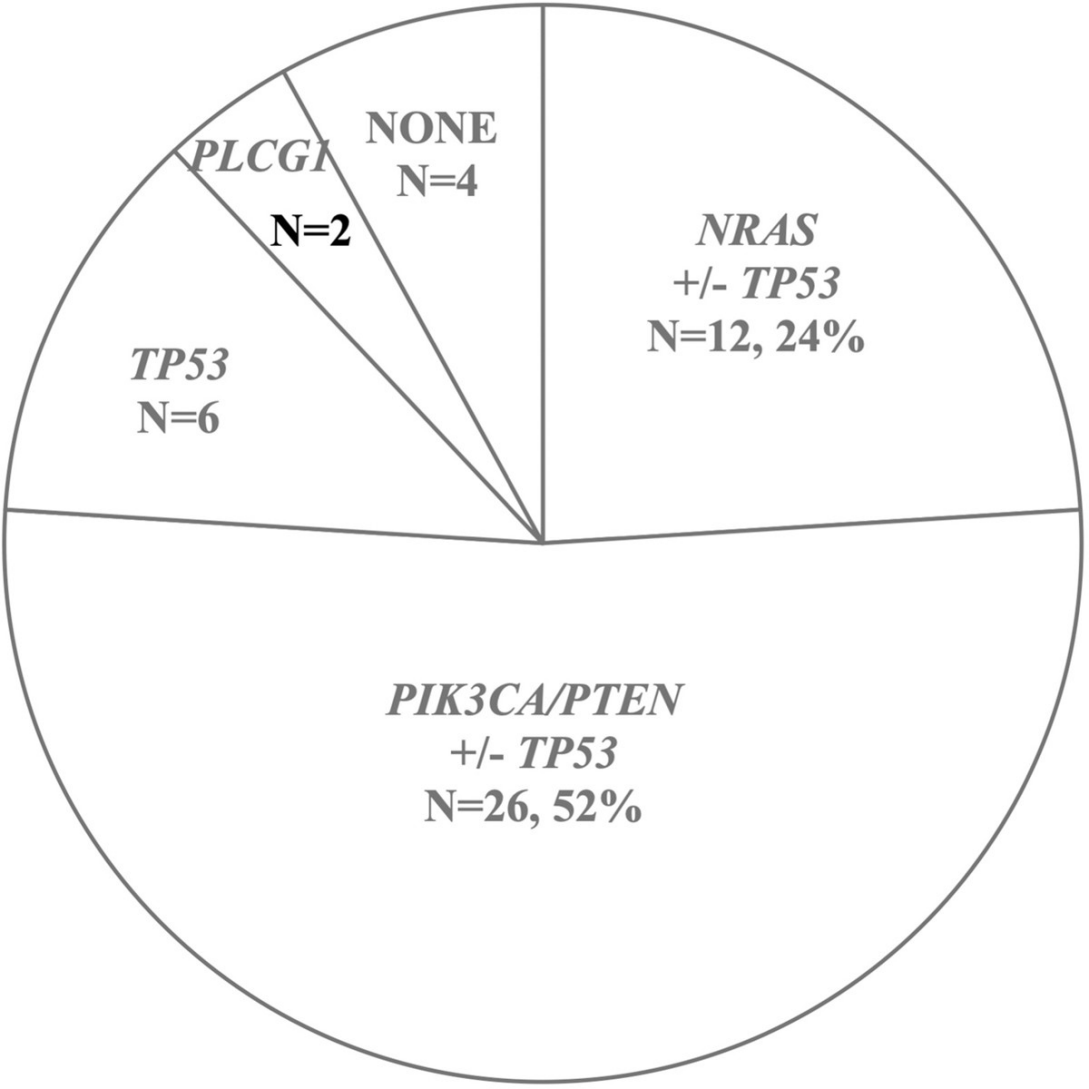
The HSA panel reveals distinct molecular subtypes of HSA distinguished by different driver mutations. Numbers and percentage of HSA cases is illustrated for each subtype.

Out of the fifty tumors sequenced so far, we were unable to identify potential driver mutations in four cases, NONE group. Thus, the HSA panel is informative in 92% (46/50) of the cases studied. Overall, the data reveal 5 distinct mutational patterns: *PIK3CA*/*PTEN*, *NRAS*, *TP53* only, *PLCG1*, and NONE group. These results suggest that the histologic diagnosis of HSA may actually represent several distinct molecular subtypes (Fig 5).

### Mutations in *PIK3CA* and *NRAS* trigger activation of downstream signaling pathways

**Fig 6:**
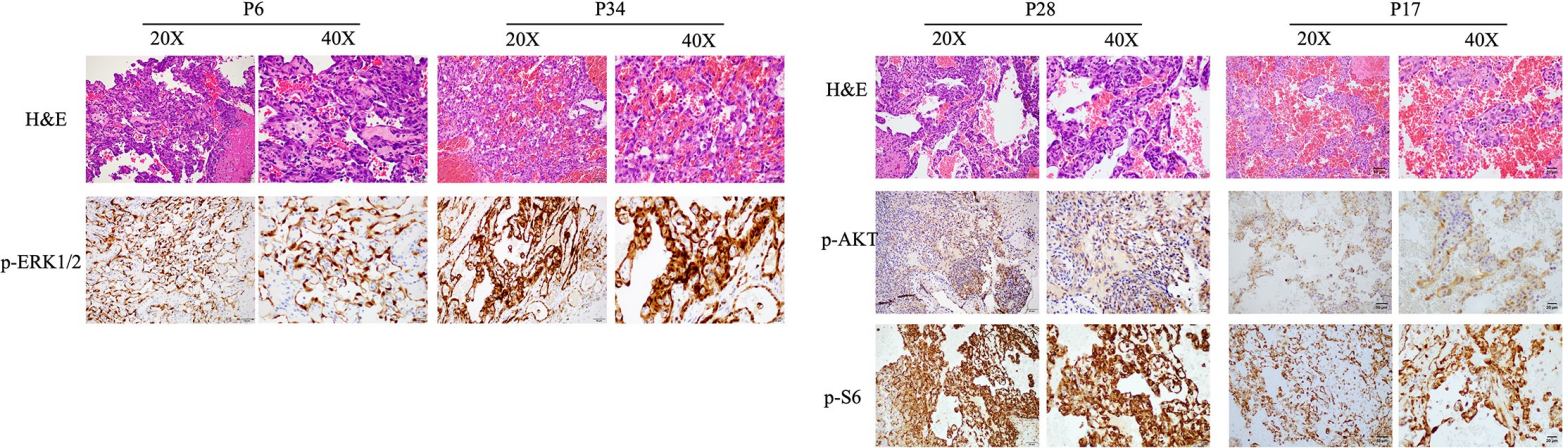
Correlation of downstream signaling with mutations in upstream genes as determined by immunohistochemistry. A: *NRAS* mutations are strongly associated with activation of MAPK signaling pathway. B: *PIK3CA*/*PTEN* mutations are strongly associated with activation of PI3K/AKT/mTOR signaling pathway.

To validate the functional relevance of the canine *PIK3CA* and *NRAS* mutants, we performed immunohistochemistry for phospho-ERK, phospho-AKT, and phopsho-S6. p-ERK is a well-established downstream effector of MAPK signaling, while p-AKT and p-S6 are classic markers of PI3K/AKT/ mTOR pathway activation [19,20]. Indeed, out of 8 *NRAS*-mutant cases that we tested by IHC, 7 showed diffuse and intense nuclear-cytoplasmic immunoreactivity for p-ERK1/2 Thr202/Tyr204 (Fig 6A). Strong p-ERK staining is observed with both *NRAS* Q61R mutant, a well-established oncogenic activating mutation, and with the previously uncharacterized *NRAS* E153K mutant. Our data suggest that *NRAS* E153K is another activating mutation that elevates MAPK/ERK signaling and contributes to sarcomagenesis. We also observed p-S6 positivity in most *NRAS* mutant cases (S2 Fig and S3 Table), consistent with previous findings that activated *NRAS* is capable of stimulating both the MAPK pathway and the PI3K pathway through activating the p110 catalytic subunit of PI3K independently of the p85 regulatory unit [21]. p-AKT Ser473 immunoreactivity is frequently observed in a wide spectrum of the *PIK3CA* and *PTEN* mutants found in our HSA cohort (Fig 6B). To confirm these results, we surveyed expression of phospho-S6 ribosomal protein Ser235/236, another indicator of activated PI3K/AKT/ mTOR signaling. p-S6 is expressed in cases with *PIK3CA* or *PTEN* mutations (Fig 6B), indicating activation of the PI3K/AKT/mTOR cascade. Our findings agree with previous studies which showed that the PI3K and MAPK pathway were frequently activated in canine HSA [20,22], and our mutational data provide molecular mechanisms for these phenotypes. IHC analysis of p-ERK, p-AKT and p-S6 in HSA cases is summarized in S3 Table.

## Discussion

### The HSA panel identifies candidate driver mutations and potentially actionable targets

High throughput DNA sequencing of tumor samples has changed the landscape of human oncology, enabling precision diagnostics to direct personalized, targeted therapy. To bring this approach to canine oncology, we developed a NGS-based targeted resequencing panel for HSA, which confirmed and extended results from 20 cases we previously studied by whole exome sequencing. The panel performed well, detecting candidate driver mutations in more than 90% of 50 clinical samples examined, with clinical information and driver genetic alterations summarized in S4 Table. Most mutations we identified correspond to well-established oncogenic mutations in humans. For example, *PIK3CA* H1047R and *PLCG1* S273F are frequently identified as activating mutations in human cancers [18,23,24]. *NRAS* Q61R is a known driver frequently found in human melanoma, angiosarcoma and other cancers [25,26]. Q61R is considered an especially aggressive activating mutant among the most common *NRAS* oncogenic mutants (G12, G13 and Q61). This may be due to severe impairment of the protein's intrinsic GTPase function, leading to accumulation of RAS-GTP and hyper-activation of RAS downstream signaling [27]. Similarly, *PLCG1* G797E corresponds to human G869E, an established gain-of-function mutant in angioimmunoblastic T cell lymphoma [9,10]. G869 is located in the SH3 domain which normally serves as an auto-inhibitory interaction interface. The G869E alteration is thought to lead to constitutive *PLCG1* activation [28]. We confirmed activation of the relevant pathways by immunohistochemistry, leading us to conclude that mutations in *NRAS* and *PIK3CA* found in HSA are indeed activating and likely are fundamental to oncogenesis. These data suggest that inhibitors specifically targeting the NRAS/MAPK or PIK3CA/PI3K pathways currently available or in human clinical trials could potentially be effective in HSA (see below for further discussion).

### Molecular subtypes defined by distinctive patterns of somatic mutations

Our analysis revealed recurrently mutated genes, recurrent mutations, and recurrent patterns of mutated targets. We sorted the latter into the following categories, [1] activating *PIK3CA* mutations, with or without *TP53* mutations (23 cases); [2] activating *NRAS* mutations, with or without *TP53* mutations (12 cases); [3] *TP53* mutations alone, with no other mutations detected (6 cases); [4] inactivating mutations in *PTEN* and *TP53* (3 cases); [5] activating *PLCG1* mutations (2 cases).

These mutually exclusive patterns of somatic driver mutations suggest that the mutational categories described above represent distinct molecular subtypes of HSA driven by different pathogenic mechanisms. We note that that inactivation of *PTEN* functions in parallel with activation of *PIK3CA*, as both events are capable of activating PI3K pathway signaling [14,29]. Thus, we combined cases with *PTEN* inactivating or *PIK3CA* activating mutations into a "PI3K pathway" group. This combined category represents the majority (52%) of cases tested. The second most prevalent category, bearing activating *NRAS* mutations, comprises 24%. *NRAS* is a critical upstream regulator of MAPK/ERK signaling cascade, whose dysregulation is frequently involved in tumorigenesis in human cancers. The remaining categories were less prevalent, and included a group bearing only *TP53* mutations (12%), *PLCG1* activating mutations (4%), and a small ‘NONE’ group with no candidate driver mutations detected (8%). Our molecular subtyping based on driver mechanisms might provide molecular explanation for some of the expression signatures previously characterized by the genome-wide RNA-seq analysis of canine visceral HSA, which identified three molecular subtypes, group1, angiogenesis; group 2, inflammation; group 3, adipogenesis [30].

The *PIK3CA/PTEN* and *NRAS* subtypes, together comprise over 75% of all HSA cases tested. Importantly, in human cancers, *PIK3CA/PTEN* mutants and *NRAS* mutants provide two distinct therapeutic targets suitable for pharmacological intervention. Numerous drugs are available to target the PI3K/AKT and MAPK/ERK pathways, including FDA approved drugs, as well as new agents being developed in preclinical settings and clinical trials [31,32]. We hypothesize that the *PIK3CA*/*PTEN* mutant and *NRAS* mutant subtypes that we have identified in HSA also represent attractive opportunities for targeted therapy in dogs, which might inform therapy in human AS. This hypothesis is supported by four observations: [1] the amino acid sequences of the mutated human and canine proteins are nearly 100% identical, and the mutations affect the same amino acids in the two species, [2] the mutations we observed generally correspond to well-known activating or inactivating oncogenic mutations in human cancers, [3] the signaling pathways involved are extremely conserved, [4] our immunohistochemical analysis provides solid evidence that these mutations are functional relevant and indeed activate downstream signaling, as they would in human cancers.

No oncogenic somatic mutations were detected in 4 cases. This could reflect the presence of somatic aberrations not detected by the sequencing methods we employed, e.g. chromosomal rearrangements, gene fusions, amplification/deletion events, and gain or loss of chromosomes. Such lesions could also be responsible for driving oncogenesis in cases in which we only detected inactivating *TP53* mutations. The inability to detect candidate driver mutations in some cases could also reflect poor DNA quality in these samples, mutations in genes not included on our panel, or mutations present at extremely low allele frequency beyond detection limit of our methods. Interestingly, some cases with only *TP53* mutations or without detectable mutations are positive for p-ERK, p-AKT or both by immunohistochemistry, possibly because of the presence of the above-mentioned alternative mechanisms, e.g. chromosomal rearrangements, gene fusions, amplification/deletion events, and gain or loss of chromosomes.

Interestingly, we also noticed breed tendencies for different mutation subtypes. PIK3CA is more prevalent in Golden retrievers, German shepherd and Labrador retrievers. While other oncogenic driver groups are less frequent in these three breeds and more sporadic in other breeds, such as Portuguese water Dog, beagle, Nova Scotia duck tolling retriever, Siberian husky, vizsla, bichon frise, etc, a conclusion cannot be drawn due to the small cohort size.

### Is canine HSA a model for human AS?

Human AS is thought to be a genetically heterogeneous group of tumors and generally features complex mutation profiles, with individual tumors harboring a wide range of genetic alterations [6,33,34]. AS is also clinically heterogeneous, with spontaneous forms as well as cases arising after therapeutic irradiation and in sun-exposed skin [35]. However, most cases of radiation-induced secondary AS bear *MYC* gene amplification, while *TP53* mutations are more common in AS arising spontaneously [33]. A subset of AS is driven by angiogenesis related gene mutations such as *PTPRB*, *PLCG1* and *KDR* (mostly in secondary cases but also found in primary cases) [6,34,36]. Activation of the MAPK/ERK pathway is also observed in some AS cases and the individual activating mutations within this pathway were often mutually exclusive, suggesting that MAPK pathway alteration is an important mechanism for AS development in these cases [33], as we have observed in HSA.

**Fig 7:**
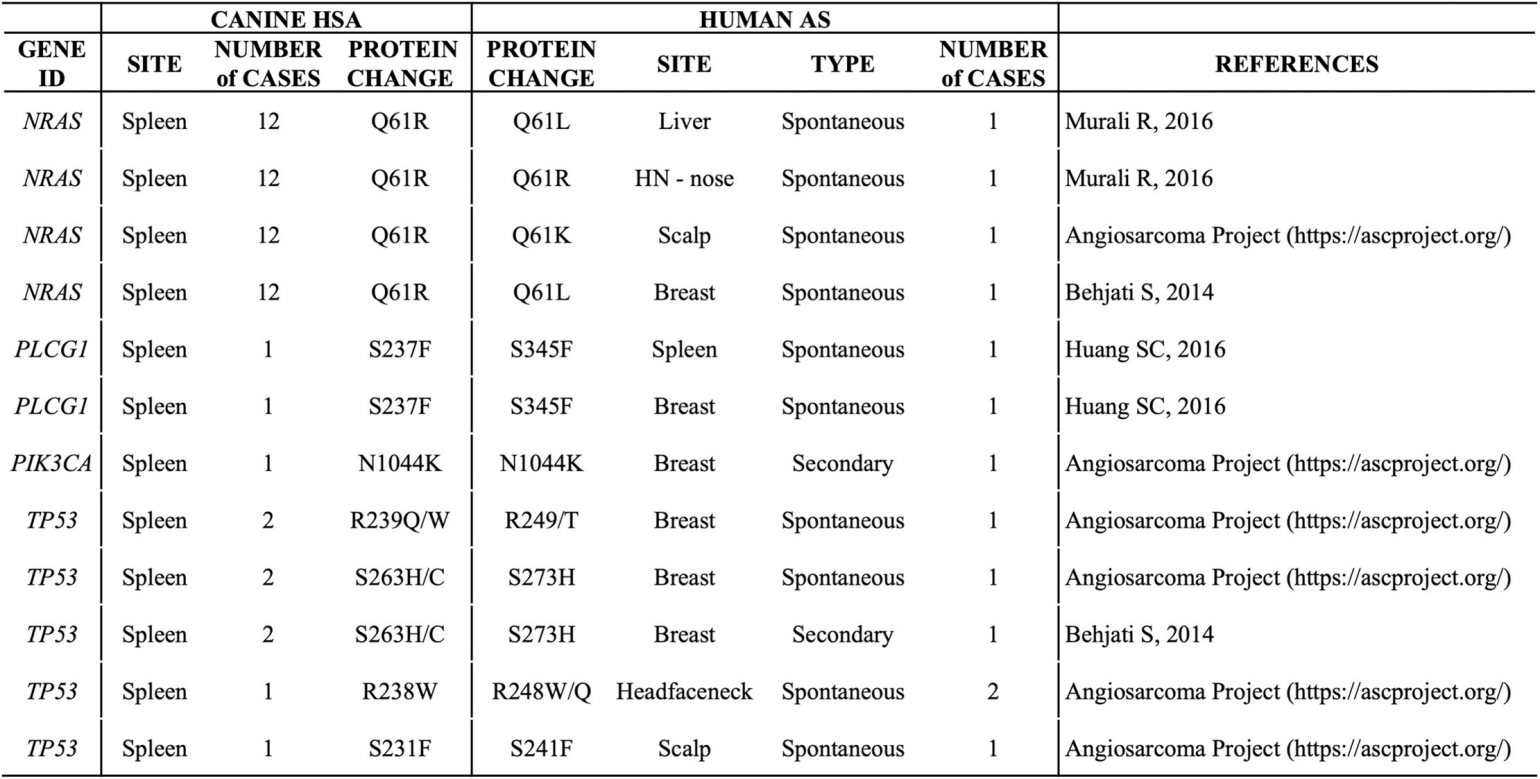
Some somatic mutations in human AS correspond to mutations at homologous positions in canine HSA.

The mutational complexity of AS is in stark contrast with the constrained mutation landscape we observe in canine HSA. This may reflect differences in the underlying pathogenesis of these diseases in the two species (sun exposure as mutagenic agent in sun-exposed AS cases, exposure to therapeutic irradiation in post-radiation AS cases, or differences in age-related mutation accumulation). Some cases of canine HSA, however, do strongly resemble human AS in the following aspects: [1] Nonsynonymous mutations in *NRAS* at position Q61, *PLCG1* (S273F, corresponding to human *PLCG1* S345F), *PIK3CA* (N1044K), and *TP53* (R248W, R280fs, S241F, R273H, etc.) are found in both canine HSA and human AS (Fig 7), [2] *TP53* is the most common mutated gene in spontaneous AS and canine HSA, [3] Alteration and activation of the MAPK pathway is a key oncogenic mechanism in both species, and [4] activation of the PI3K/AKT/mTOR pathway is observed in a subset of human AS (42%) [37]. This is consistent with the prevalent activation of PI3K/AKT pathway in canine HSA indicated by our sequencing data and IHC results, suggesting another common pathogenic mechanism shared between human AS and canine HSA (Fig 7).

Overall, the presence of homologous mutations and the conservation of key signaling pathways that are activated indicates that canine HSA mirrors some cases of human AS. Our findings here suggest that canine HSA could serve as an appropriate model to understand the pathogenesis of certain cases of AS, to evaluate prognostic biomarkers, and to explore novel treatment strategies by testing targeted agents in client-owned dogs diagnosed with HSA. The results of such trials might inform human trial design and translate effective therapies to human AS that harbor similar genetic/pathway alterations.

### Diagnostic utility of the HSA panel

As far as we are aware, this is one of the first applications of a NGS panel approach, commonly used in human cancer diagnostics, in canine cancer. NGS panels provide several advantages in analysis of clinical cancer samples (formalin fixed, paraffin embedded), including high accuracy and high sensitivity, as sufficient sequencing depth is important for assessing clinical tumor samples in which tumor cells are mixed with varying amounts of normal stromal tissue and inflammatory cells. Other features important for clinical utility include turnaround time and cost, both of which are advantages of panel testing. Further work is needed to validate the clinical utility of the biomarkers provided by the HSA panel.

## Methods

### HSA tissue collection

Formalin fixed paraffin embedded (FFPE) blocks of fifty HSA tumor and matched normal tissues were selected at the biopsy service in the Penn Vet Diagnostic Laboratory. Residual (left-over) tissues after diagnosis were collected and used for this study; therefore, no IACUC protocol was required. H&E microscopic slides were reviewed and confirmed by a veterinary pathologist (ACD); tumor and normal tissues for dissection were identified and circled. FFPE tissue cores were harvested from tumor and normal blocks using a 1.0 mm punch tool.

### HSA panel targeted sequencing

Genomic DNA (gDNA) was extracted from FFPE tissue cores using GeneRead DNA FFPE Kit (Qiagen). Quality and quantity of gDNA were assessed with the TapeStation 4200 system and the Qubit 2.0 Fluorometer, following manufacturer’s protocol. Oligos targeting genomic region of interest were designed on Design Studio and were synthesized and pooled into Custom Amplicon Tubes (CATs) (Illumina). Dual pool design generates two CATs, CAT-A and CAT-B. Two amplicon libraries were prepared for each gDNA using CAT-A or CAT-B, following Truseq Custom Amplicon (TSCA) Low Input workflow (Illumina) and were then sequenced on a Miniseq sequencer (Illumina). Sequence data were analyzed with the local run manager (LRM) software. Specifically, the Amplicon DS analysis module was selected, allowing for read alignment against reference genomic region specified in the manifest file and the generation of the vcf file containing somatic variants including both single nucleotide variants (SNVs) and small insertions/deletions (indels). Variants were called from each pool separately, then cross-compared and combined into a single vcf file. The variants passed quality control if they met the following criteria: 1) present in both pools, 2) cumulative depth of 1000X or an average depth of 500X per pool, 3) variant frequency of >3% as reported in the merged VCF file.

SnpEff (version: 4.2) was used to annotate the variants for gene information and amino acid changes, as well as to predict the functional impacts of the changes, e.g. low, high or moderate. Variants that passed quality control were selected for subsequent manual review, pathway analysis and further annotation. Additionally, filtering was performed to exclude synonymous, intronic, and untranslated region variants except for splice site variants. In addition to the 20 matched normal controls used in our previous study [5], 10 additional matched normal DNA samples (normal tissue from 10 patients) were subjected to TSCA dual-pool library preparation, sequencing and variant calling. Variants found in these 30 normal tissue samples were filtered out from the final variant list.

### Immunohistochemistry staining

5 μm thick paraffin sections were mounted on ProbeOn^™^ slides (Thermo Fisher Scientific). The immunostaining procedure was performed using a Leica BOND RXm automated platform combined with the Bond Polymer Refine Detection kit (Leica). Briefly, after dewaxing and rehydration, sections were pretreated with the epitope retrieval BOND ER2 high pH buffer (EDTA Based pH = 9.0) (20 min, 95°C). Endogenous peroxidase was inactivated with 3% H2O2 (10 min, RT). Nonspecific protein-protein interactions were blocked with Leica PowerVision IHC/ISH Super Blocking solution (PV6122) for 30 minutes at RT. Rabbit monoclonal primary antibodies against Phospho-AKT Ser473 (CST #4060), Phospho-ERK1/2 Thr202/Tyr204 (CST #4370) and Phospho-S6 Ribosomal Protein Ser235/236 (CST #4858) were used at dilutions of 1:300 and 1:500, respectively. All the antibodies were prepared using the SignalStain Antibody Diluent (CST #8112) and incubated on the sections for 45 minutes at RT. A biotin-free polymeric IHC detection system consisting of HRP conjugated anti-rabbit IgG was then applied for 25 minutes at RT. Immunoreactivity was revealed with the diaminobenzidine (DAB) chromogen reaction. Slides were finally counterstained in hematoxylin, dehydrated in an ethanol series, cleared in xylene, and permanently mounted with a resinous mounting medium (Thermo Scientific ClearVueTM coverslipper). Sections of selected human melanomas previously confirmed to be positive for the different phosphorylated targets were included as positive controls in each run. Two pathologists (ACD and XX) examined each immunostaining result and were blinded from the genetic information.

## Supporting information

S1 Fig Examples of mean sequencing coverage of HSA-panel. X-axis: sample ID, y-axis: sequencing coverage.
(DOCX)

S2 Fig Both PI3K and MAPK signaling pathways are activated in NRAS mutant cases as indicated by positive staining of pS6 and pERK. (DOCX)

S1 Table Mutations details in PIK3CA in canine HSA cohort. (DOCX)

S2 Table Mutations details in TP53 in canine HSA cohort. (DOCX)

S3 Table Summary of IHC analysis in HSA cases harboring distinct candidate driver mutations. (DOCX)

S4 Table Clinical features of canine HSA and candidate driver mutations. (DOCX)
