# Supplementary material for: Molecular subtypes in canine hemangiosarcoma reveal similarities with human angiosarcoma
Source: PLoS One. 2020 Mar 25;15(3):e0229728. doi: 10.1371/journal.pone.0229728 (PMC7094861; doi:10.1371/journal.pone.0229728)
Supplement: S1 Fig — X-axis: sample ID, y-axis: sequencing coverage. (DOCX) [file pone.0229728.s001.docx]

Supplementary Fig. S1


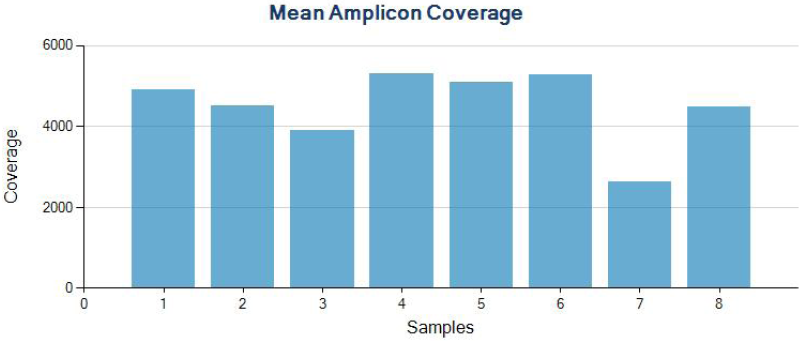


Supplemental Fig S1: Examples of mean sequencing coverage of HSA-panel. X-axis: sample ID, y-axis: sequencing coverage.
