# Supplementary figures and images for: Molecular subtypes in canine hemangiosarcoma reveal similarities with human angiosarcoma
Source: PLoS One. 2020 Mar 25;15(3):e0229728. doi: 10.1371/journal.pone.0229728 (PMC7094861; doi:10.1371/journal.pone.0229728)

Supplementary Fig. S2


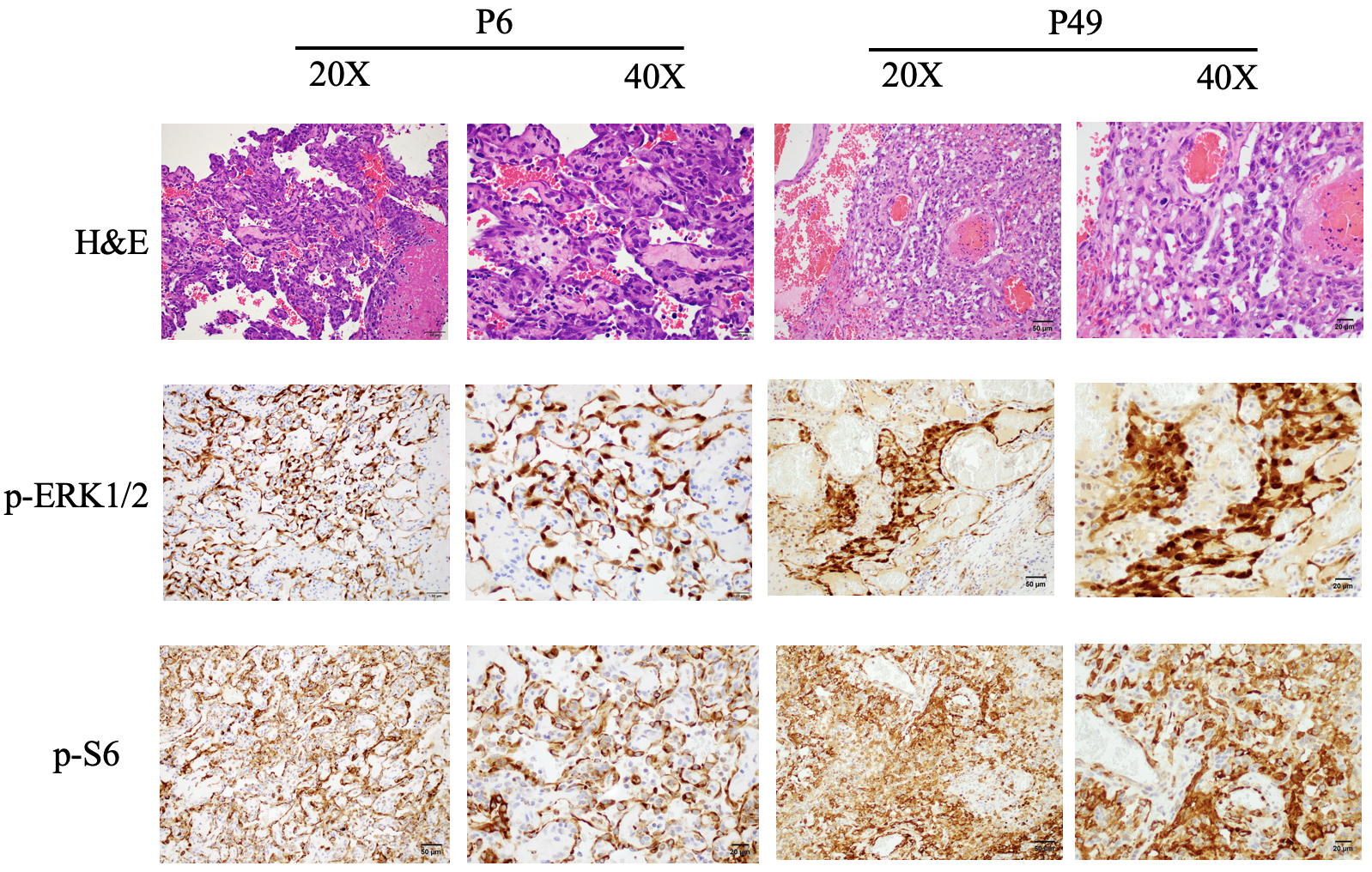

Supplement: S2 Fig — (DOCX) [file pone.0229728.s002.docx]
