# Supplementary material for: Molecular subtypes in canine hemangiosarcoma reveal similarities with human angiosarcoma
Source: PLoS One. 2020 Mar 25;15(3):e0229728. doi: 10.1371/journal.pone.0229728 (PMC7094861; doi:10.1371/journal.pone.0229728)
Supplement: S1 Table — (DOCX) [file pone.0229728.s003.docx]

Supplementary Table S1: Mutations details in PIK3CA in canine HSA cohort

| **Gene ID** | **Sample ID** | **Protein**  **change** | **Mutation**  **type** | **Chromosome** | **Start**  **position** | **End**  **position** | **Reference**  **Allele** | **Variant**  **Allele** |
| --- | --- | --- | --- | --- | --- | --- | --- | --- |
| PIK3CA | P32 | Asn1044Lys | Missense | chr34 | 12675666 | 12675666 | T | A |
| PIK3CA | P4 | Asp350Gly | Missense | chr34 | 12651135 | 12651135 | A | G |
| PIK3CA | P39 | Cys420Arg | Missense | chr34 | 12660730 | 12660730 | T | C |
| PIK3CA | P12PT | Glu726Lys | Missense | chr34 | 12666386 | 12666386 | G | A |
| PIK3CA | P13 | Glu726Lys | Missense | chr34 | 12666386 | 12666386 | G | A |
| PIK3CA | P36 | Gly1049Arg | Missense | chr34 | 12675679 | 12675679 | G | C |
| PIK3CA | P48 | Gly1049Arg | Missense | chr34 | 12675679 | 12675679 | G | C |
| PIK3CA | P10PT | His1047Arg | Missense | chr34 | 12675674 | 12675674 | A | G |
| PIK3CA | P11 | His1047Arg | Missense | chr34 | 12675674 | 12675674 | A | G |
| PIK3CA | P15 | His1047Arg | Missense | chr34 | 12675674 | 12675674 | A | G |
| PIK3CA | P16 | His1047Arg | Missense | chr34 | 12675674 | 12675674 | A | G |
| PIK3CA | P17 | His1047Arg | Missense | chr34 | 12675674 | 12675674 | A | G |
| PIK3CA | P22 | His1047Arg | Missense | chr34 | 12675674 | 12675674 | A | G |
| PIK3CA | P29 | His1047Arg | Missense | chr34 | 12675674 | 12675674 | A | G |
| PIK3CA | P3 | His1047Arg | Missense | chr34 | 12675674 | 12675674 | A | G |
| PIK3CA | P30 | His1047Arg | Missense | chr34 | 12675674 | 12675674 | A | G |
| PIK3CA | P35 | His1047Arg | Missense | chr34 | 12675674 | 12675674 | A | G |
| PIK3CA | P37 | His1047Arg | Missense | chr34 | 12675674 | 12675674 | A | G |
| PIK3CA | P38 | His1047Arg | Missense | chr34 | 12675674 | 12675674 | A | G |
| PIK3CA | P7 | His1047Arg | Missense | chr34 | 12675674 | 12675674 | A | G |
| PIK3CA | P20 | His1047Leu | Missense | chr34 | 12675674 | 12675674 | A | T |
| PIK3CA | P23 | His1047Leu | Missense | chr34 | 12675674 | 12675674 | A | T |
| PIK3CA | P51 | Thr1025Ala | Missense | chr34 | 12675607 | 12675607 | A | G |
