# Supplementary material for: Molecular subtypes in canine hemangiosarcoma reveal similarities with human angiosarcoma
Source: PLoS One. 2020 Mar 25;15(3):e0229728. doi: 10.1371/journal.pone.0229728 (PMC7094861; doi:10.1371/journal.pone.0229728)
Supplement: S2 Table — (DOCX) [file pone.0229728.s004.docx]

Supplementary Table S2: Mutations details in TP53 in canine HSA cohort

| **Gene ID** | **Sample ID** | **Canine**  **Protein Change** | **Human**  **Protein change** | **Mutation**  **type** | **Chromosome** | **Start**  **position** | **Reference**  **Allele** | **Variant**  **Allele** |
| --- | --- | --- | --- | --- | --- | --- | --- | --- |
| TP53 | P30 | Leu27_splice | L25_splice | Splice_Site | chr5 | 32565070 | C | T |
| TP53 | P7 | Gly177_splice | G187_splice | Splice_Site | chr5 | 32563877 | C | G |
| TP53 | P23 | Glu214_splice | E224_splice | Splice_Site | chr5 | 32563683 | C | A |
| TP53 | P19 | Leu100* | L111* | Nonsense_Mutation | chr5 | 32564610 | A | T |
| TP53 | P39 | Arg186* | R196* | Nonsense_Mutation | chr5 | 32563770 | G | A |
| TP53 | P48 | Arg296* | R306* | Nonsense_Mutation | chr5 | 32562957 | G | A |
| TP53 | P7 | Pro141fs | P152fs | Frame_Shift_Del | chr5 | 32563984 | TG | T |
| TP53 | P38 | Arg270fs | R280fs | Frame_Shift_Del | chr5 | 32563038 | CG | C |
| TP53 | P29 | Tyr317fs | Y327fs | Frame_Shift_Del | chr5 | 32562601 | GTGAAATA | G |
| TP53 | P33 | Gly256Arg | G266R | Missense_Mutation | chr5 | 32563077 | C | T |
| TP53 | P32 | Val161Asp | V172D | Missense_Mutation | chr5 | 32563925 | A | T |
| TP53 | P28 | Arg263Cys | R273C | Missense_Mutation | chr5 | 32563056 | G | A |
| TP53 | P36 | His168Gln | H179Q | Missense_Mutation | chr5 | 32563903 | A | T |
| TP53 | P34 | Arg239Gln | R249Q | Missense_Mutation | chr5 | 32563388 | C | T |
| TP53 | P40 | Gly256Glu | G266E | Missense_Mutation | chr5 | 32563076 | C | T |
| TP53 | P45 | Gly256Glu | G266E | Missense_Mutation | chr5 | 32563076 | C | T |
| TP53 | P49 | Cys265Gly | C275G | Missense_Mutation | chr5 | 32563050 | A | C |
| TP53 | P49 | Arg257His | R267H | Missense_Mutation | chr5 | 32563073 | C | T |
| TP53 | P51 | Arg263His | R273H | Missense_Mutation | chr5 | 32563055 | C | T |
| TP53 | P10PT | Arg272His | R282H | Missense_Mutation | chr5 | 32563028 | C | T |
| TP53 | P11 | Arg272His | R282H | Missense_Mutation | chr5 | 32563028 | C | T |
| TP53 | P33 | Arg272His | R282H | Missense_Mutation | chr5 | 32563028 | C | T |
| TP53 | P26 | Arg327His | R337H | Missense_Mutation | chr5 | 32562199 | C | T |
| TP53 | P29 | Val162Leu | V173L | Missense_Mutation | chr5 | 32563923 | C | A |
| TP53 | P37 | Val162Leu | V173L | Missense_Mutation | chr5 | 32563923 | C | A |
| TP53 | P18T | Val264Leu | V274L | Missense_Mutation | chr5 | 32563053 | C | G |
| TP53 | P10PT | Pro268Leu | P278L | Missense_Mutation | chr5 | 32563040 | G | A |
| TP53 | P11 | Pro268Leu | P278L | Missense_Mutation | chr5 | 32563040 | G | A |
| TP53 | P31 | Glu160Lys | E171K | Missense_Mutation | chr5 | 32563929 | C | T |
| TP53 | P15 | Ser231Phe | S241F | Missense_Mutation | chr5 | 32563412 | G | A |
| TP53 | P25 | Leu320Pro | L330P | Missense_Mutation | chr5 | 32562598 | A | G |
| TP53 | P27 | Arg163Trp | R174W | Missense_Mutation | chr5 | 32563920 | G | A |
| TP53 | P4 | Arg163Trp | R174W | Missense_Mutation | chr5 | 32563920 | G | A |
| TP53 | P18T | Arg192Trp | R202W | Missense_Mutation | chr5 | 32563752 | G | A |
| TP53 | P3 | Arg238Trp | R248W | Missense_Mutation | chr5 | 32563392 | G | A |
| TP53 | P35 | Arg239Trp | R249W | Missense_Mutation | chr5 | 32563389 | G | A |
| TP53 | P16 | Cys124Tyr | C135Y | Missense_Mutation | chr5 | 32564036 | C | T |
| TP53 | P17 | Cys228Tyr | C238Y | Missense_Mutation | chr5 | 32563421 | C | T |
| TP53 | P31 | Asp271Tyr | D281Y | Missense_Mutation | chr5 | 32563032 | C | A |
| TP53 | P52 | Thr72Pro | Not conserved | Missense_Mutation | chr5 | 32564695 | T | G |
