# Supplementary material for: Molecular subtypes in canine hemangiosarcoma reveal similarities with human angiosarcoma
Source: PLoS One. 2020 Mar 25;15(3):e0229728. doi: 10.1371/journal.pone.0229728 (PMC7094861; doi:10.1371/journal.pone.0229728)
Supplement: S3 Table — (DOCX) [file pone.0229728.s005.docx]

Supplementary Table S3: Summary of IHC analysis in HSA cases harboring distinct candidate driver mutations

| **Sample ID** | **Mutatons** | **p-ERK** | **p-AKT** | **p-S6** |
| --- | --- | --- | --- | --- |
| P17 | PIK3CA/TP53 | - | + | + |
| P36 | PIK3CA/TP53 | - | + | + |
| P7 | PIK3CA/TP53 | NA | + | + |
| P39 | PIK3CA/TP53 | NA | NA | + |
| P23 | PIK3CA/TP53 | + | + | + |
| P30 | PIK3CA/TP53 | NA | NA | + |
| P38 | PIK3CA/TP53 | - | + | + |
| P18 | PTEN/TP53 | - | NA | + |
| P28 | PTEN/TP53 | - | + | + |
| P31 | TP53/TP53 | + | + | + |
| P6 | NRAS | + | + | + |
| P47 | NRAS(Glu153Lys) | + | + | + |
| P49 | NRAS/TP53/TP53 | + | + | + |
| P34 | NRAS/TP53 | + | + | + |
| P26 | NRAS/TP53 | + | NA | NA |
| P24 | NRAS | + | NA | NA |
| P1 | NRAS | + | NA | NA |
| P9 | NRAS | - * | NA | NA |
