# Supplementary material for: Molecular subtypes in canine hemangiosarcoma reveal similarities with human angiosarcoma
Source: PLoS One. 2020 Mar 25;15(3):e0229728. doi: 10.1371/journal.pone.0229728 (PMC7094861; doi:10.1371/journal.pone.0229728)
Supplement: S4 Table — (DOCX) [file pone.0229728.s006.docx]

Supplementary Table S4: Clinical features of canine HSA and candidate driver mutations

| **Sample ID** | **Organ** | **Breed** | **Age** | **Sex** | **Mutation** |
| --- | --- | --- | --- | --- | --- |
| P1 | Spleen | Shep Mix | 13y | MC | NRAS |
| P3 | Spleen | Nova Scotia Duck Tolling Retriever | 11y | FS | PIK3CA/TP53 |
| P4 | Spleen | Beagle Mix | 9y | MC | PIK3CA/TP53 |
| P5 | Spleen | Rottweiler Mix | 10y | MC | PLCG1 |
| P6 | Spleen | Lab | 10y | MC | NRAS |
| P7 | Spleen | Lab | 9y | MC | PIK3CA/TP53 |
| P8 | Spleen | Soft-coated Wheaten terrier | 12y | MC | PLCG1 |
| P9 | Spleen | Not specified | 7y | FS | NRAS |
| P10PT | Spleen | Golden Retriever | 10.5y | M | PIK3CA/TP53 |
| P10MT | Omentum | Golden Retriever | 10.5y | M | PIK3CA/TP53 |
| P12PT | Spleen | German Shepherd | 8y | FS | PIK3CA |
| P12MT | Liver | German Shepherd | 8y | FS | PIK3CA |
| P15 | Spleen | Golden Retriever | 12y | MC | PIK3CA/TP53 |
| P16 | Spleen | Portugese Water Dog | 7y | M | PIK3CA |
| P17 | Spleen | Lab | 10.5y | MC | PIK3CA/TP53 |
| P18 | Spleen | Lab | 10.5y | MC | PTEN/TP53 |
| P19 | Spleen | Bichon Frise | 12y | FS | PTEN/TP53 |
| P20 | Spleen | German Shepherd | 10.5y | F | PIK3CA |
| P21 | Spleen | Lab | 8y | MC |  |
| P22 | Spleen | Golden Retriever | 9y | MC | PIK3CA/TP53 |
| P23 | Spleen | Golden Retriever | 11.5y | MC | PIK3CA/TP53 |
| P24 | Spleen | English Springer Spaniel | 9y | FS | NRAS |
| P25 | Spleen | Mixed Breed | 15Y | MC | TP53 |
| P26 | Spleen | Golden Retriever | 8y | FS | NRAS/TP53 |
| P27 | Spleen | Boxer | 10.5y | FS | TP53 |
| P28 | Spleen | Border Collie | 9y | MC | TP53 |
| P29 | Spleen | Cocker Spaniel/Poodle | 12y | MC | PIK3CA/TP53 |
| P30 | Spleen | Vizsla | 9.5y | FS | PIK3CA/TP53 |
| P31 | Spleen | German Shepherd | 13y | FS | TP53/TP53 |
| P32 | Spleen | German Shepherd | 8y | FS | PIK3CA/TP53 |
| P33 | Spleen | Pit Bull | 8y | M | TP53/TP53 |
| P34 | Spleen | Lab | 9y | MC | NRAS/TP53 |
| P35 | Spleen | German Shepherd | 12y | MC | PIK3CA/TP53 |
| P36 | Spleen | Mixed | 13y | FS | PIK3CA/TP53 |
| P37 | Spleen | Golden Retriever | 8y | MC | PIK3CA/TP53 |
| P38 | Spleen | German Shepherd | 8y | F | PIK3CA/TP53 |
| P39 | Spleen | Lab | 8y | FS | PIK3CA/TP53 |
| P40 | Spleen | German Shepherd | 8y | MC | PTEN/TP53 |
| P41 | Spleen | French Bulldog | 10y | FS | NRAS |
| P42 | Spleen | Mastiff | 8y | FS | NRAS |
| P43 | Spleen | Golden Retriever | 9y | MC | NONE |
| P44 | Spleen | Mixed | 12y | MC | NONE |
| P45 | Spleen | Belgian Sheepdog | 8y | MC | NRAS/TP53 |
| P46 | Spleen | Bichon Frise | 9y | MC | NONE |
| P47 | Spleen | Mixed | 11y | MC | NRAS |
| P48 | Spleen | Lab | 9y | MC | PIK3CA/TP53 |
| P49 | Spleen | Miniature Schnauzer | 12y | MC | NRAS/TP53/TP53 |
| P50 | Spleen | Shiba Inu | 12y | M | NRAS |
| P51 | Spleen | Siberian Husky | 11y | FS | PIK3CA/TP53 |
| P52 | Spleen | Cockapoo | 12y | MC | TP53 |

MC: Male Castrated, FS: Female Spayed, M: Intact Male, F: Intact Female
